# Virtual Life Story Club Intervention to Improve Loneliness and Apathy in Community-Dwelling Older Adults: Protocol for a Mixed Methods Feasibility Study

**DOI:** 10.2196/70518

**Published:** 2025-06-04

**Authors:** Chava Pollak, Helena M Blumen, Lily Zhou, Jennifer Wong, Ying Jin, Atul Bhattiprolu, Sophie Anfang, Mirnova E Ceïde

**Affiliations:** 1 Department of Neurology Stony Brook Medicine Stony Brook, NY United States; 2 Life Story Club Brooklyn, NY United States; 3 Department of Psychiatry and Behavioral Sciences Albert Einstein College of Medicine Bronx, NY United States

**Keywords:** apathy, cognition, community-dwelling, loneliness, reminiscence therapy

## Abstract

**Background:**

Reminiscence therapy is a noninvasive, nonpharmacological intervention that has been shown to improve cognition, mood, functional status, quality of life, and apathy in older adults. Group reminiscence therapy combines structured social engagement and recounting of personal stories that address both social connection (a risk factor for cognitive decline) and cognition. Life Story Club is an established, nonprofit organization that provides virtual group reminiscence therapy for older adults to reduce loneliness and promote a sense of belonging and has not been formally studied.

**Objective:**

This study aims to explore the feasibility of a Life Story Club intervention to improve loneliness and apathy in community-dwelling older adults.

**Methods:**

A prospective, single-arm, single-center, pilot study will be conducted to compare loneliness and apathy in 50 lonely individuals without dementia at baseline who receive a virtual group reminiscence therapy intervention. The intervention will be delivered weekly over 12 weeks. Loneliness will be assessed with the UCLA Loneliness Scale and apathy will be assessed with the Apathy Evaluation Scale before and after the intervention. Feasibility will be assessed using quantitative and qualitative measures including feasibility of screening and enrollment, acceptability, and program satisfaction. Qualitative interviews will be conducted with a subset of 30 individuals to explore acceptability, barriers, and facilitators of the intervention.

**Results:**

The proposed study is funded by a pilot grant from the Institute for Clinical and Translational Research at Albert Einstein College of Medicine. Recruitment and data collection are planned for July 2025.

**Conclusions:**

This study will provide evidence for the feasibility of virtual group reminiscence therapy for community-dwelling older adults to reduce loneliness and apathy. Our approach is both innovative and pragmatic because we will leverage an existing community-based service, with an established infrastructure and track record within the community to deliver the intervention. As such, the proposed research has the potential for broad implications for community-based research and aligns with multiple translational science principles.

**International Registered Report Identifier (IRRID):**

PRR1-10.2196/70518

## Introduction

### Background

An estimated 6.9 million US older adults are living with Alzheimer disease and related dementias (ADRD) and the incidence increases with age [1]. ADRD is characterized by a progressive loss of cognition and function that eventually results in loss of independence and mortality. Currently, there are no curative treatments for ADRD. Thus, there is increased focus on nonpharmacological interventions that target modifiable risk factors for cognitive decline and dementia in older adults.

Apathy is a psychological syndrome characterized by a lack of motivation and interest, flattening of affect, and social withdrawal that predicts cognitive decline and increases the risk of ADRD by 20%-70% [2-10]. The prevalence of apathy ranges between 20% and 30% in community-dwelling older adults [11]. Apathy predicts the incidence of predementia syndromes mild cognitive impairment (MCI) and motoric cognitive risk syndrome, which is characterized by subjective cognitive complaints and slow gait [12,13]. Apathy is associated with social isolation related to a lack of interest in engagement [14]. Similarly, loneliness is highly prevalent in community-dwelling older adults (up to 43% [15]) and is associated with an increased risk of cognitive decline and ADRD [16,17]. Apathy and loneliness both represent potentially modifiable risk factors for cognitive decline and dementia and reminiscence therapy (RT) has been studied to target both these risk factors [18-20].

**Figure 1:**
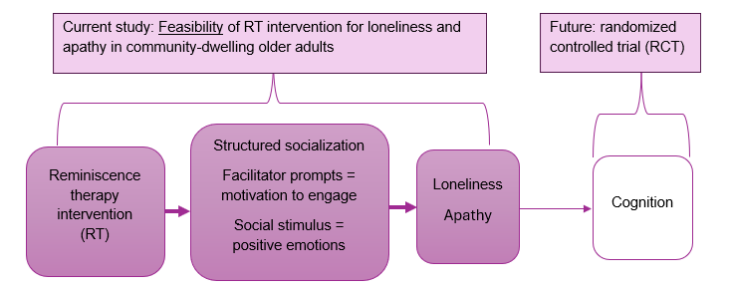
Conceptual model of a reminiscence therapy intervention for loneliness and apathy and mechanisms for how the intervention may work. The left side of the figure represents the current proposal where we propose to investigate the feasibility of reminiscence therapy for loneliness and apathy. This research will inform and support a future randomized controlled trial of a reminiscence therapy intervention to improve cognition.

RT is a noninvasive, nonpharmacological intervention that has been shown to improve cognition, mood, functional status, quality of life, and apathy in older adults [18-24]. RT uses life story recollection in individual or group settings to improve psychological well-being. Prompts, photos, or music are all techniques used to trigger reminiscence, and the intervention can be delivered in person or virtually. Mechanisms for RT for apathy work through structured socialization with prompting from facilitators that stimulate motivation to engage. In addition, social stimuli lead to positive emotions through the sharing of personal stories. Sharing personal stories and a structured social environment that provides opportunities for meaningful engagement that may not happen in a casual social context address loneliness as well. A conceptual model of RT to address loneliness and apathy and how reminiscence may work as an intervention is visually depicted in Figure 1.

Cognitive decline in aging is multifactorial with multiple possible causes and there is significant variability in cognition based on the presence or absence of risk factors such as social factors (eg, loneliness) and apathy. In addition, the multiple aspects of social relationships (eg, meaning and purpose in life and social support) and psychological well-being (eg, depression) influence cognition via multiple possible paths. Meaning and purpose in life were associated with cognitive decline, and incident MCI and Alzheimer disease in prior studies [25]. In addition, studies investigating volunteering interventions to provide meaningful social, physical, and cognitive activities for older adults reduced brain aging [26]. RT is similarly organized to provide structured social engagement and stimulate cognitive activity through exercising autobiographical memory [27] and improvements in cognition may be mediated through improved life meaning and purpose. Depression is a well-known risk factor for cognitive decline [28] and loneliness and the relationship between loneliness and depression may be bidirectional [29]. In addition, apathy and depression are distinct neuropsychiatric conditions that often co-occur and symptoms may overlap [30]. Given the mechanisms of RT are not well understood, multiple aspects of social relationships and psychological well-being may be considered as mediators of the relationship between RT and health outcomes.

A growing body of research on RT for older adults targets individuals with ADRD and older adults in long-term care, as well as healthy older adults living in the community [18,20,21,31]. In addition, individual RT applied using virtual reality was shown to improve cognition and psychological well-being in institutionalized older adults, compared to traditional RT [32]. Studies on the effects of a virtually delivered group reminiscence therapy intervention that combines the benefits of a group intervention and is delivered virtually using communication technologies are scarce. The novelty of this proposal is that the intervention is designed, developed, and delivered via an established community-based organization by nonclinicians and has not been formally studied.

### Life Story Club

Life Story Club (LSC) is an established, nonprofit organization founded in Brooklyn, New York, in 2019. The organization is dedicated to reducing loneliness in older adults through RT and has already successfully served more than 1500 older adults. LSCs are virtual, no-cost RT clubs that meet weekly. The clubs are led by trained facilitators who propose 2 story prompts per meeting to invite participants to share their stories (eg, “As a child, what did you say you wanted to be when you grew up?” Or, “Tell a story about a time you or your loved one didn’t accept defeat.”). Participants alternate responding to prompts and sharing their stories. Facilitators are nonclinicians with teaching, arts, or social science backgrounds. Facilitators undergo comprehensive training including shadowing, on-the-job learning, and certification in Mental Health First Aid and SAFECare LGBTQ+ (lesbian, gay, bisexual, transgender, queer/questioning, and others) Cultural Competency Training prior to leading a club. LSC serves roughly 400 older adults per year and clubs are offered in English, Spanish, Mandarin, or Cantonese. In addition, groups can be phone or video-based to provide for broader access. Clubs can also be tailored based on particular interests such as LGBTQ+ clubs or cooking clubs.

Weekly sessions follow a structured format to ensure consistency and engagement. Weekly prompts are sent to participants via email, text, or phone 1-2 days before the meeting. Sessions begin with greetings and icebreakers. The facilitator then introduces the prompt and shares an example story. Each participant shares a 5-minute story related to one of the prompts and participants are encouraged to engage with the storyteller’s experience. Sessions conclude with final thoughts. LSC prompts are drawn from a library designed to evoke a range of autobiographical memories and reflections. Each week, prompts rotate across categories for broad depth and variety in storytelling experiences. LSC structure and prompt categories are detailed in Table 1.

**Table 1:** Life Story Club program structure.

Parameters		Descriptions
**Weekly schedule**		
	Weeks 1-2	Introductory sessions with get-to-know-you prompts.
	Weeks 3-7	Sessions tailored to participants’ interests; participants take turns sharing stories based on weekly prompts.
	Week 8	Dedicated recording session where stories are recorded for archival purposes.
	Weeks 9-11	Regular storytelling sessions continue.
	Week 12	Special event for unveiling of the group’s Life Story Library.
**Prompt categories and examples**		
	Cultural and historical memories	What is a historical “first” you’ve lived through?
	Occasion-based memories	Can you tell us about a memorable celebration or gathering?
	Location-based memories	Tell us about a meaningful or memorable hangout spot in your life.
	Youth and childhood	Who was your childhood best friend?
	Career-based memories	Can you tell us about an extraordinary day at work?
	Current and future life	Can you share a story about a recent triumph?
	Relationships	What was something passed down to you from a family member, friend, or mentor?
	Identity and self-reflection	When was a time you changed your mindset or way of thinking?

Individuals are referred to LSC via primary care providers and through community outreach via naturally occurring retirement communities, public libraries, and older adult centers. Caregivers, family members, or individuals can also self-refer via the LSC website. RT as delivered by LSC can potentially improve accessibility and equity given its virtual delivery in multiple languages and can extend the reach of the intervention to homebound and rural older adults—the population who are at the highest risk for cognitive decline, tend to be hard to reach, and often not included in research for those reasons. LSC is a well-established community-based organization with a formalized infrastructure to serve community-based older adults.

### Study Objectives

The purpose of this study will be to explore the feasibility of a virtual group reminiscence therapy (vGRT) intervention delivered by LSC to improve loneliness and apathy in community-dwelling older adults. Rather than developing a new intervention, we propose to capitalize on an existing community-based resource to deliver a novel, virtual RT intervention directly to individuals in the community. We hypothesize that a virtual, group RT intervention will be feasible and acceptable for community-dwelling older adults.

## Methods

### Study Design

This study is a prospective, nonrandomized, mixed methods feasibility study testing the impact of the LSC intervention. The purpose of the proposed study design is to test the feasibility of the intervention and provide preliminary data for a larger randomized controlled trial (RCT).

### Recruitment

Participants will be recruited via partnerships with local community-based organizations serving older adults, home care agencies, as well as referrals from health care teams within the health system (eg, post-acute care managers, primary care social workers, and providers). Retention will be maximized by using preintervention counseling, regular opportunities for questions and feedback from participants, and compensation for completing pretest assessments, posttest assessments, and qualitative interviews at costs consistent with Institutional Review Board guidelines. All participants who participated in the LSC intervention will be invited to participate in qualitative interviews and we will enroll up to 30 participants or until saturation is reached.

#### Inclusion and Exclusion Criteria

Inclusion criteria are (1) age 60 and older and (2) ability to speak sufficient English or Spanish to complete assessments. These criteria are established to ensure that participants can fully engage with the study materials and accurately report their experiences. Individuals with dementia based on the AD8 or previous diagnosis of dementia, severe auditory or visual loss, and lack of access to either an internet connection or telephone will be excluded. The AD8 is an 8-item cognitive screening test sensitive to detecting early cognitive changes associated with common dementias including Alzheimer disease; scores of 2 or more indicate cognitive impairment [33] and can be completed via telephone.

#### Intervention

LSC is a virtual, social storytelling space for older adults aged 60 and older where members can share life stories and connect with other members to promote a sense of belonging, purpose, and meaning. Clubs include up to 15 members that meet once a week for 1 hour. The number of participants per group is determined based on past levels of participation where 10-15 individuals are recruited per group and 6-8 attend each session due to the nature of the population (eg, multimorbidity and acute illness). The protocol for the study will not deviate from routine LSC protocols and will be offered similarly to the usual schedule and prompts as described in the introduction except for dedicated recording sessions which will be replaced by a usual session (Table 1). Clubs are led by a nonclinician facilitator who offers prompts to trigger discussion and reminiscence. Sessions begin with greetings after which 2 prompts are offered by the facilitator. The facilitator manages the group to encourage participation by all members. To promote technology equity (colloquially referred to as “techquity”), LSC has been delivered via live video or telephone according to participant preference to promote technology inclusiveness. Participants for this study will have the option to use video or telephone based on preference. The primary endpoint for this study will be 3 months based on durations of other RT interventions [31,34]. We will additionally collect assessment data at 6 months to preliminarily determine the feasibility and efficacy of a 6-month intervention in participants who continue to participate in the intervention over a longer period. Fidelity will be assessed via periods of observation by research staff to observe if components of the intervention are met (eg, 2 prompts per session and individual participation).

### Data Collection

Assessments will be collected via telephone at baseline, 3 months, and 6 months post RT. Participants will receive a gift card for each completed assessment. Qualitative interviews will be conducted with a subset of 30 participants to assess satisfaction with the intervention, what aspects can be improved, and explore barriers and facilitators of participation, including digital literacy. A semistructured interview guide is included in Multimedia Appendix 1. Interviews will take place at an agreed-upon location over 60 minutes and will be conducted by the primary investigator or coinvestigator. We will collect data on the following outcome measures and covariates:

### Primary Outcome

Our primary outcomes will be loneliness and apathy. This is based on our hypothesis that targeting these cognitive decline and dementia risk factors will result in improved cognition in the longer term. Given this is a feasibility study and 3- and 6-month intervals may be too short of a time frame to see a difference in cognition and that the study will not be powered to detect differences in cognition, we propose to examine differences in loneliness and apathy, risk factors for cognitive decline, which can plausibly change in this short interval.

#### UCLA Loneliness Scale

Loneliness will be assessed using the UCLA Loneliness Scale [35]. The UCLA Loneliness Scale is a well-validated and widely used tool designed to measure subjective feelings of loneliness and social isolation. It evaluates the overall sense of loneliness through 20 items that reflect social isolation and dissatisfaction with one’s social relationships [35]. Participants will be asked to rate each item on a 4-point Likert scale, ranging from 1 (never) and 2 (rarely) to 3 (sometimes) and 4 (often), with higher scores indicating greater loneliness. The total score will be derived by summing the responses across all items, with possible scores ranging from 20 to 80. In this study, loneliness will be treated as a continuous variable. The UCLA Loneliness Scale demonstrates high internal consistency and strong construct validity, correlating well with other measures of loneliness and social well-being [35].

#### Apathy Evaluation Scale

Apathy will be assessed using the Apathy Evaluation Scale (AES), a validated 18-item tool designed to measure the cognitive, behavioral, and emotional aspects of apathy [36]. Items are scored on a 4-point Likert scale, and higher scores indicate greater levels of apathy. The AES shows high internal consistency and good validity, with strong correlations to other measures of mood and motivation, distinguishing apathy from related conditions such as depression [36].

### Secondary Outcomes

Secondary outcomes will be examined as potential mediators of effects between RT, loneliness, and apathy.

#### Cognition

Cognition will be measured by the telephone Montreal Cognitive Assessment (T-MoCA). The T-MoCA is a validated tool designed to screen for MCI across domains such as attention, memory, language, visuospatial skills, executive function, and orientation [37,38]. Adapted from the original Montreal Cognitive Assessment for remote administration, the T-MoCA is particularly useful for populations where in-person assessments may be challenging. The T-MoCA consists of 22 items and participants will be scored out of 22 points, with a score below 18 typically indicating cognitive impairment. The T-MoCA has shown good reliability and validity that is concurrent with the original in-person Montreal Cognitive Assessment [37].

#### PROMIS: Meaning and Purpose

Meaning and purpose in life will be assessed using the PROMIS (Patient-Reported Outcomes Measurement Information System) Meaning and Purpose Scale. The PROMIS Meaning and Purpose Scale is a validated tool that measures an individual’s sense of purpose, significance, and meaning in life. This scale will assess the extent to which individuals feel their lives are filled with meaning, direction, and goals, contributing to overall well-being and mental health. Participants will respond to a series of items on a 5-point Likert scale, entailing 1 (never), 2 (rarely), 3 (sometimes), 4 (often), and 5 (always), with higher scores indicating a stronger sense of meaning and purpose. PROMIS provides a reliable and valid measure of meaning in life, demonstrating internal consistency across diverse populations [39]. It also shows strong correlations with other related constructs, such as life satisfaction and psychological well-being, reinforcing its construct validity [39].

#### Geriatric Depression Scale

Depressive symptoms will be assessed using the Geriatric Depression Scale–Short Form (GDS-SF) [40]. The GDS-SF is a widely validated screening tool for depressive symptoms in older adults. It is a shortened version of the original 30-item GDS and contains 15 dichotomous (yes or no) questions designed to assess mood and affect, with a focus on identifying symptoms of depression in the older adult population [40]. Scores range from 0 to 15, with higher scores indicating greater depressive symptoms. The GDS-SF is validated in multiple populations, with good internal consistency and strong concurrent validity when compared to other depression scales such as the Hamilton Depression Rating Scale (HDRS) [41].

#### MOS Social Support Scale

Social support will be assessed using the Medical Outcomes Study (MOS) Social Support Survey [42]. The MOS Social Support Scale is a widely used 19-item instrument designed to measure various dimensions of social support, including emotional, informational, tangible, affectionate, and positive social interaction. Items are rated on a 5-point Likert scale ranging from 1 (none of the time) to 5 (all of the time), with higher scores indicating greater perceived social support. The MOS Social Support Scale was validated across diverse populations, demonstrating high internal consistency and strong construct validity, with robust correlations to other measures of social functioning and mental health [42].

#### Covariates

Covariates were selected based on their reported associations with cognition, social connection, and overall health. Covariate data will be used to describe the population engaged in the study and will be informative in terms of feasibility and generalizability. Age will be recorded in years, calculated from the self-reported date of birth, as age influences cognitive decline [43]. Gender will be categorized as male or female based on self-report, considering that gender differences influence cognitive performance and vulnerability to cognitive decline [44]. Race will be self-reported and classified into categories to explore potential disparities in health outcomes, as prior studies highlight the role of race in health inequalities. Education, assessed in years of formal schooling completed, will be collected through self-report, as increased educational attainment is associated with better cognitive function and social engagement in older adults [45]. Participants will also self-report their occupation before retirement, recognizing that work-related activities can provide cognitive stimulation and social interaction, which impact mental health outcomes. Marital status will be evaluated at baseline, with responses categorized as never married, married, widowed, divorced, or separated, given that marital status is linked to social support and well-being, crucial for cognitive health [46]. Living arrangements will be documented to differentiate those living alone, with family, or in institutional settings, as prior studies suggest that living arrangements can affect loneliness and access to social networks [47]. Social determinants of health including transportation and housing will be assessed via self-report. The FRAIL Scale will be used to assess frailty through self-reported items related to fatigue, resistance, ambulation, illness, and weight loss, recognizing that frailty is associated with cognitive decline and increased risk for adverse health outcomes in older adults [48] and both loneliness and apathy are associated with frailty and functional decline [49,50]. Finally, overall health and comorbidities will be self-reported and supplemented by information from the electronic health record, encompassing chronic conditions such as diabetes, hypertension, and cardiovascular diseases, which have known associations with cognitive impairment and social functioning.

#### Feasibility Outcomes

Feasibility will be determined by screening, enrollment, and acceptability of the intervention. Acceptability of the intervention will also be assessed via program satisfaction surveys (eg, satisfaction, mood, belonging, connection with members) at 3 months and 6 months as well as qualitative interviews to explore program acceptability, barriers and facilitators of participation, and technology accessibility. Outcome measures including feasibility and health outcomes are detailed in Textbox 1 and Table 2. Benchmarks are determined by prior LSC metrics and expectations for recruitment, enrollment, and participation based on prior LSC participants. The metrics we propose are based on study-specific retention efforts (eg, preintervention counseling, relationship building with participants, and participant incentives).

**Feasibility outcomes.**

**Criteria**

- Screening
- Enrollment
- Acceptability
- Program evaluation
- Barriers and facilitators
- Technology access

**Benchmarks**

- No. of individuals screened/total referred (≥70% good)
- No. of individuals who agree/total screened (≥70 good)
- No. of individuals who complete 9/12 visits/total enrolled (≥70 good)
- Survey
- Open-ended interview
- Open-ended interview

**Table 2:** Outcome measures.

Outcome measures			Assessment		Metrics
**Primary outcomes**					
	Loneliness	UCLA Loneliness Scale		Likert scale 1-4 (1=never, 4 often). Total from a set of 20 items	
	Apathy	Apathy Evaluation Scale		18 items on 4-point Likert scale Higher scores=increased apathy	
**Secondary outcomes**					
	Cognition	T-MoCA^a^		Global cognition; ≤26=MCI	
	Meaning and purpose	PROMIS-meaning and purpose		4 items; higher scores=higher meaning or purpose	
	Depressive symptoms	Geriatric Depression Scale		15 items; Score of ≥5 suggests depression	
	Social support	MOS Social Support Scale		19 items; perceived social support; higher scores=greater support	
	Physical function	FRAIL Scale		5 items; self-reported fatigue, resistance, ambulation, illness, weight loss	
	Overall health	Global health status		Self-reported physician diagnosis of 9 common conditions	

^a^T-MoCA: telephone Montreal Cognitive Assessment; version 8.1 to be administered at baseline, version 8.2 at 3 months, and version 8.3 at 6 months to reduce practice effect.

Covariates/Demographics to be collected: age, gender, race/ethnicity, education, occupation before retirement, marital status, living arrangements, and social determinants of health (Montefiore version).

### Data Analysis

The primary purpose of this exploratory project is to test the feasibility of an LSC intervention and to provide estimates of the efficacy of the LSC intervention to inform a future RCT. We will enroll a total sample size of 50 participants and expect an estimated 15% dropout rate. This sample size follows guidelines for pilot studies and is appropriate for exploring feasibility [51]. Descriptive statistics will be used to report sample characteristics, feasibility markers, and measures. Statistical analyses will be conducted using Stata (version 18.5; StataCorp LLC). Repeated-measures ANOVA will be applied to test for overall differences in loneliness and apathy at baseline, 3 months, and 6 months. Secondary outcomes will be analyzed for differences in trends before and after the intervention and associations with changes in loneliness and apathy. We will test whether these measures mediate changes in loneliness and apathy from baseline using linear regression models.

We will use field notes and memos throughout the qualitative interview process to explore qualitative data. Data will be deductively analyzed using NVivo 15 software.

### Data Management and Monitoring

Data will be entered and stored in a secure, HIPAA (Health Insurance Portability and Accountability Act)-compliant REDCap (Research Electronic Data Capture; Vanderbilt University) database. Only study staff tasked with data entry and analysis will have access to the data. No identifying information will be written or transcribed for this study.

### Ethical Considerations

This study is being conducted in accordance with the Declaration of Helsinki ethics code. The protocol will be reviewed by the Albert Einstein College of Medicine institution review board. Because the intervention poses no more than minimal risk, a waiver of written consent was requested. Oral informed consent will be sought via telephone prior to study enrollment and prior to qualitative interviews.

## Results

The proposed research is funded by a pilot grant from the Institute for Clinical and Translational Research at Albert Einstein College of Medicine. Recruitment and data collection for the proposed research are planned for July 2025.

## Discussion

This study will yield feasibility data on the use of a vGRT intervention to improve loneliness and apathy in community-dwelling older adults without dementia and a qualitative assessment of the intervention. Our protocol is unique in that the proposed study will provide preliminary data for a novel, vGRT to improve loneliness and apathy in community-dwelling older adults. The intervention has the potential to improve accessibility and equity given its virtual delivery in multiple languages and can potentially reach homebound and rural older adults—populations who are at the highest risk for cognitive decline, tend to be hard to reach and are often not included in research for those reasons. Our approach is both innovative and pragmatic because we will leverage an existing community-based service, with an established infrastructure and track record within the community to deliver the intervention, rather than developing a new intervention in a research setting. As such, the proposed research has the potential for broad implications for community-based research, and data can be informative for community-engaged aging research. Moreover, the intervention is scalable for older adults across cultures and geographic locations given the intervention is virtual and can be tailored based on the interests of participants.

The study has potential limitations. As a pilot study, the primary purpose of the proposed study is to test the feasibility of vGRT and provide preliminary efficacy data to improve loneliness and apathy. The pilot will include a single-arm and participants will not be randomized. The study will lay the groundwork for a future RCT of a LSC intervention to improve cognition in older adults. LSC engagement to date is consistent with prior work that has shown older adults can be exceptionally adherent to technological interventions with thoughtful planning and troubleshooting [52]. It is possible that technology access varies across populations and may affect the recruitment of diverse participants. We plan to collect quantitative and qualitative data regarding technology access which will inform future studies of LSC interventions. Future studies might also explore the provision of technology (eg, a tablet to allow access to videoconferencing software) to further promote access and participation. Currently, LSC is limited to 4 languages (English, Spanish, Cantonese, and Mandarin), which may exclude participants who speak other languages. In addition, the current study is restricted to individuals with and those without cognitive impairment and excludes individuals with dementia based on the AD8 assessment. The feasibility of LSC in its current format of virtual delivery, in a medium-sized group setting, with a nonclinical facilitator in individuals with advanced dementia remains an area of opportunity for future research.

In summary, this study will provide valuable data for a future RCT of a vGRT intervention to improve loneliness and apathy in community-dwelling older adults. If successful, the intervention can be widely scaled as a nonpharmacological intervention to prevent cognitive decline and promote social engagement in aging. The proposed methods to leverage community-based partnerships to establish and deliver effective interventions within communities can serve as a model for more community-engaged aging research.

## Appendix

Multimedia Appendix 1 Additional material including the interview guide and consent form.

## Abbreviations

ADRD: Alzheimer disease and related dementias
AES: Apathy Evaluation Scale
GDS-SF: Geriatric Depression Scale–Short Form
HDRS: Hamilton Depression Rating Scale
HIPAA: Health Insurance Portability and Accountability Act
LGBTQ+: lesbian, gay, bisexual, transgender, queer/questioning, and others
LSC: Life Story Club
MCI: mild cognitive impairment
MOS: Medical Outcomes Study
PROMIS: Patient-Reported Outcomes Measurement Information System
RCT: randomized controlled trial
REDCap: Research Electronic Data Capture
RT: reminiscence therapy
T-MoCA: telephone Montreal Cognitive Assessment
vGRT: virtual group reminiscence therapy

## Data Availability

The datasets generated or analyzed during this study will be available from the corresponding author on reasonable request.
