# Supplementary material for: Virtual Life Story Club Intervention to Improve Loneliness and Apathy in Community-Dwelling Older Adults: Protocol for a Mixed Methods Feasibility Study
Source: JMIR Res Protoc. 2025 Jun 4;14:e70518. doi: 10.2196/70518 (PMC12177422; doi:10.2196/70518)
Supplement: Multimedia Appendix 1 [file resprot_v14i1e70518_app1.docx]

**Supplementary Material**

**Appendix A. Semi-structured Interview Guide**

**Appendix B. Oral Consent for LSC Intervention**

**Appendix C. Oral Consent for Qualitative Interview**

**Appendix A. Participant Perspectives of the LSC Intervention: Semi-Structured Interview Guide**

1. What does a typical day for you look like?
   1. How were your LSC days different than your typical day?
2. What made you decide to join the LSC club?
   1. How did you find out about the club?
3. How did you participate in LSC – phone or video?
   1. What do you think about participating in a social club via phone or video?
   2. How do you think the phone/video affected your participation?
   3. How did having options for participation influence your decision to join?
4. What did you think about the LSC?
   1. What did you most enjoy?
   2. What do you think can be improved?
5. What did you most enjoy talking about during the sessions?
   1. What made it enjoyable?
   2. Were there any prompts you didn’t enjoy?
6. What role did the facilitator play in managing the meeting?
   1. How did you feel about the time you had to share your story?
   2. What do you think is the best way to share stories?
7. How did participating in the club affect your health?
8. How did participating in the club affect your social life?
   1. Did participating in the club influence your social life outside of the weekly sessions? If so, how?
   2. How do you feel about your social life now compared to before you joined?

General prompts will include, “tell me more, “why do you think that is?”, “what did (or would) you do next?” etc.

**Appendix B. ORAL CONSENT SCRIPT FOR** **VIRTUAL GROUP REMINISCENCE THERAPY (Participant)**

**Hello.** My name is **[name],** and I am from **the Albert Einstein College of Medicine.** We would like to ask a few questions about yourself that will us understand how our program is affecting your health and well-being. You do not have to participate; it is your choice. Your decision will not affect your rights or benefits or your access to care.

If you say yes, we will ask you some questions about yourself and about your health before you start the Lifestory Club program. We will contact you over the telephone or via video conference. This will take approximately 30-60 minutes. During the interview, we will ask you questions about your physical health and well-being. We will ask you the same questions every 3 months after you start the program.

**Would you like to complete this part of the project?**

**____Yes**

**____No**

You may be uncomfortable answering some questions. You do not have to answer all the questions and you may stop at any time.

A risk of taking part in this study is the possibility of a loss of confidentiality or privacy. Loss of privacy means having your personal information shared with someone who is not on the study team and was not supposed to see or know about your information. The study team plans to protect your privacy.

You may or may not receive personal and direct benefit for participating in this study. The possible benefits of participating include helping to improve the quality of the program to assist individuals who have concerns about loneliness or cognition.

Some researchers may develop tests, treatments or products that are worth money. You will not receive payment of any kind for your information or for any tests, treatments, products, or other things of value that may result from the research.

We will pay you $25 for completing each interview with a $25 gift card.

There will be an audio voice recording of the interview that will only be used for transcribing responses to the interview questions. You will not receive any monetary compensation for allowing yourself to be recorded. The tapes will be destroyed at the end of the study.

We will do our best to keep your information safe by using a special code. We do not plan to share the information from this study with other researchers. The researchers and study staff follow federal and state laws to protect your privacy.

Your information and research records will be kept confidential. Your study information will be kept if it is useful for the research described in this form.

The only people who can see your research records are:

- Researchers and other individuals who work with the researchers.
- Organizations and institutions involved in this research, including those that fund the research, if applicable.
- Groups that review research such as central reviewers, Institutional Review Boards, the Office for Human Research Protections, the US Food and Drug Administration, data coordinating centers, and domestic and foreign agencies that regulate research.

The purposes of these uses and disclosures are to (1) conduct the study and (2) make sure the study is being done correctly. The information covered under this form may no longer be protected by federal privacy laws (such as HIPAA) once disclosed, and those persons who receive your health information may share your information with others without your additional permission. All these groups have been asked to keep your information confidential.

Information from this study may be used in future research studies by our study team.

There are several people and groups who may see your study information to make sure the study is being done correctly. This includes:

- the research team and staff who work with them
- the organization that funded the research
- groups that review research such as the Einstein IRB, and the Office for Human Research Protections

These people who receive your health information may not be required by privacy laws to protect it and may share your information with others without your permission, if permitted by laws governing them. All these groups have been asked to keep your information confidential.

If you change your mind and don’t want your information used for the study anymore, you can call the person in charge of this study. Her name is **Dr. Mirnova Ceide,** and she can be reached on **718-430-3808**. Or you can call Einstein Institutional Review Board at 718-430-2253. They will let you know how to write to the Principal Investigator to tell him you want to stop participating. Just remember, if we have already used your information for the study, the use of that information cannot be cancelled.

Do you have any questions? You may ask me now or contact **Dr. Mirnova Ceide** about your questions or problems with this study.

| **CONSENT TO PARTICIPATE** | | |
| --- | --- | --- |
| _________________________ |  | _________ |
| Printed name of participant |  | Date |
|  |  |  |
| _________________________ |  | _________ |
| Printed name of proxy |  | Date |
| _________________________ | ________________________________________ | _________ |
| Printed name of the person conducting the consent process | Signature | Date |

**Appendix C. ORAL CONSENT SCRIPT FOR QUALITATIVE INTERVIEW**

**Hello.** My name is **[name],** and I am from **the Albert Einstein College of Medicine,** and I would like to hear about your experience with the Lifestory Club. We would like to ask a few questions regarding your time at the club to help us improve the quality of the program. You do not have to participate; it is your choice. Your decision will not affect your rights or benefits or your access to care.

If you say yes, we will ask you some questions pertaining to your experience with the group and of interacting with our team. We will contact you to answer some questions over the telephone or via video conference. This will take approximately 30-60 minutes. During the interview, we will ask you questions about your experience with the loneliness assessment that you completed.

**Would you like to complete this part of the project?**

**____Yes**

**____No**

You may be uncomfortable answering some questions. You do not have to answer all the questions and you may stop at any time.

A risk of taking part in this study is the possibility of a loss of confidentiality or privacy. Loss of privacy means having your personal information shared with someone who is not on the study team and was not supposed to see or know about your information. The study team plans to protect your privacy.

You may or may not receive personal and direct benefit for participating in this study. The possible benefits of participating include helping to improve the quality of the program to assist individuals who have concerns about loneliness or cognition.

Some researchers may develop tests, treatments or products that are worth money. You will not receive payment of any kind for your information or for any tests, treatments, products, or other things of value that may result from the research.

We will pay you $25 for completing the interview with a $25 gift card.

There will be an audio voice recording of the interview that will only be used for transcribing responses to the interview questions. You will not receive any monetary compensation for allowing yourself to be recorded. The tapes will be destroyed at the end of the study.

We will do our best to keep your information safe by using a special code. We do not plan to share the information from this study with other researchers. The researchers and study staff follow federal and state laws to protect your privacy.

Your information and research records will be kept confidential. Your study information will be kept if it is useful for the research described in this form.

The only people who can see your research records are:

- Researchers and other individuals who work with the researchers.
- Organizations and institutions involved in this research, including those that fund the research, if applicable.
- Groups that review research such as central reviewers, Institutional Review Boards, the Office for Human Research Protections, the US Food and Drug Administration, data coordinating centers, and domestic and foreign agencies that regulate research.

The purposes of these uses and disclosures are to (1) conduct the study and (2) make sure the study is being done correctly. The information covered under this form may no longer be protected by federal privacy laws (such as HIPAA) once disclosed, and those persons who receive your health information may share your information with others without your additional permission. All these groups have been asked to keep your information confidential.

Information from this study may be used in future research studies by our study team.

There are several people and groups who may see your study information to make sure the study is being done correctly. This includes:

- the research team and staff who work with them
- the organization that funded the research
- groups that review research such as the Einstein IRB, and the Office for Human Research Protections

These people who receive your health information may not be required by privacy laws to protect it and may share your information with others without your permission, if permitted by laws governing them. All these groups have been asked to keep your information confidential.

If you change your mind and don’t want your information used for the study anymore, you can call the person in charge of this study. Her name is **Dr. Mirnova Ceide,** and she can be reached on **718-430-3808**. Or you can call Einstein Institutional Review Board at 718-430-2253. They will let you know how to write to the Principal Investigator to tell him you want to stop participating. Just remember, if we have already used your information for the study, the use of that information cannot be cancelled.

Do you have any questions? You may ask me now or contact **Dr. Mirnova Ceide** about your questions or problems with this study.

| **CONSENT TO PARTICIPATE** | | |
| --- | --- | --- |
| _________________________ |  | _________ |
| Printed name of participant |  | Date |
|  |  |  |
| _________________________ |  | _________ |
| Printed name of proxy |  | Date |
| _________________________ | ________________________________________ | _________ |
| Printed name of the person conducting the consent process | Signature | Date |
